# Polyamine biosynthesis is critical for growth and differentiation of the pancreas

**DOI:** 10.1038/srep13269

**Published:** 2015-08-24

**Authors:** Teresa L. Mastracci, Morgan A. Robertson, Raghavendra G. Mirmira, Ryan M. Anderson

**Affiliations:** 1 Department of Pediatrics, Indiana University School of Medicine, USA; 2 Department of Physiology, Indiana University School of Medicine, USA; 3 Center for Diabetes and Metabolic Diseases, Indiana University School of Medicine, USA

## Abstract

The pancreas, in most studied vertebrates, is a compound organ with both exocrine and endocrine functions. The exocrine compartment makes and secretes digestive enzymes, while the endocrine compartment, organized into islets of Langerhans, produces hormones that regulate blood glucose. High concentrations of polyamines, which are aliphatic amines, are reported in exocrine and endocrine cells, with insulin-producing β cells showing the highest concentrations. We utilized zebrafish as a model organism, together with pharmacological inhibition or genetic manipulation, to determine how polyamine biosynthesis functions in pancreatic organogenesis. We identified that inhibition of polyamine biosynthesis reduces exocrine pancreas and β cell mass, and that these reductions are at the level of differentiation. Moreover, we demonstrate that inhibition of ornithine decarboxylase (ODC), the rate-limiting enzyme in polyamine biosynthesis, phenocopies inhibition or knockdown of the enzyme deoxyhypusine synthase (DHS). These data identify that the pancreatic requirement for polyamine biosynthesis is largely mediated through a requirement for spermidine for the downstream posttranslational modification of eIF5A by its enzymatic activator DHS, which in turn impacts mRNA translation. Altogether, we have uncovered a role for polyamine biosynthesis in pancreatic organogenesis and identified that it may be possible to exploit polyamine biosynthesis to manipulate pancreatic cell differentiation.

The growth and development of a multicellular organism necessitates the successive differentiation of specialized cell types from pools of undifferentiated progenitor cells. This coordinated process ultimately results in the specification of tissues composed of distinct cell types that harmonize to produce functioning organs; each distinct cell type must pass through several semi-differentiated pluripotent cell populations between the zygote and mature organism. Deciphering the mechanisms that direct this cell-specific differentiation is a key biological question with important ramifications for understanding the pathogenesis or treatment of many diseases, including diabetes mellitus.

Diabetes is a syndrome that results from the destruction or dysfunction of the insulin-producing β cells in the pancreas^1^. In all organisms, β cell development begins during embryogenesis. Pancreatic progenitor cells are specified in the endoderm following the reception of secreted signals. These secreted signals then initiate a cascade of pancreas-specific transcription factors within the pancreatic progenitor cells^2^. In particular, these progenitor cells first co-express the transcription factors pancreatic and duodenal homeobox 1 (Pdx1) and pancreas transcription factor 1a (Ptf1a), and subsequently differentiate into cells composing all pancreatic lineages: exocrine, endocrine and duct cells^3^,^4^,^5^. However before differentiation occurs, the progenitor cells increase in number and spatially organize into an intricate epithelial tree; progenitors in the trunk domain upregulate Neurogenin3 (Neurog3) before differentiating into endocrine cells^6^,^7^, whereas those in the tip domain express CarboxypeptidaseA (Cpa1) and are first multipotent before becoming pro-acinar cells^8^. Decades of research have revealed a complex transcription factor cascade that drives pancreas cell lineage specification and differentiation^2^. Many laboratories are utilizing this knowledge to create protocols to generate insulin-expressing β-like cells *in vitro* for therapeutic purposes^9^,^10^,^11^,^12^,^13^,^14^,^15^. Although some success has been achieved, our understanding of the factors required for β cell development remains incomplete. Clearly looking beyond transcription factor regulation is necessary for continued advancement.

Interestingly, high concentrations of polyamines have been reported in both exocrine and endocrine pancreatic cells, with the insulin-producing β cell showing the highest concentration among the islet cells^16^,^17^. Polyamines (putrescine, spermidine, and spermine) are polycationic, low molecular weight aliphatic amines that are required for cellular proliferation^18^. The first step in the biosynthesis of polyamines requires the rate-limiting enzyme ornithine decarboxylase (ODC), whose sole function is to catalyze the decarboxylation of ornithine to putrescine^19^. Disruption of the gene encoding ODC (*Odc1*) results in early embryonic lethality in the mouse^20^. Although *Odc1*-null blastocysts can be recovered, their development beyond embryonic day (E) 3.5 is halted by widespread cellular apoptosis and embryo death^20^, providing clear evidence that polyamine biosynthesis is critical for embryogenesis. Moreover, this early requirement for polyamines in embryogenesis has, to date, precluded the study of polyamine biosynthesis during pancreatic organogenesis.

In this study, using the versatile zebrafish as a model organism, we demonstrate that although the multipotent pancreatic progenitor population appears unchanged in size, altering polyamine biosynthesis through pharmacologic inhibition of ODC affects both exocrine cells and β cells at the level of differentiation. In addition, we uncover that this pancreatic requirement for polyamine biosynthesis is connected to the process of mRNA translation; inhibition of the downstream enzyme deoxyhypusine synthase (DHS), which catalyzes the posttranslational modification of the eukaryotic initiation factor 5A (eIF5A), phenocopies the inhibition of polyamine biosynthesis. Ultimately, our study identifies that it may be possible to exploit polyamine biosynthesis to manipulate pancreatic cell growth and differentiation.

## Results

### Inhibition of polyamine biosynthesis alters exocrine pancreas growth

Ornithine decarboxylase (Odc1; ODC) is the rate-limiting enzyme required for polyamine biosynthesis. The drug difluoromethylornithine (DFMO; Eflornithine) specifically inhibits ODC thereby inhibiting polyamine biosynthesis^21^. Using protein sequence alignment (ClustalW^22^), zebrafish (*Danio rerio*) Odc1 was determined to be 75% identical to the mouse (*Mus musculus*) and human (*Homo sapiens*) orthologs (Supplementary Fig. S1). Thus, we took advantage of transgenic zebrafish lines to analyze how pancreas development was altered following pharmacological inhibition of ODC; the use of DFMO bypassed the early embryonic requirement for ODC^20^ and permitted the examination of alterations during organogenesis.

**Figure 1:**
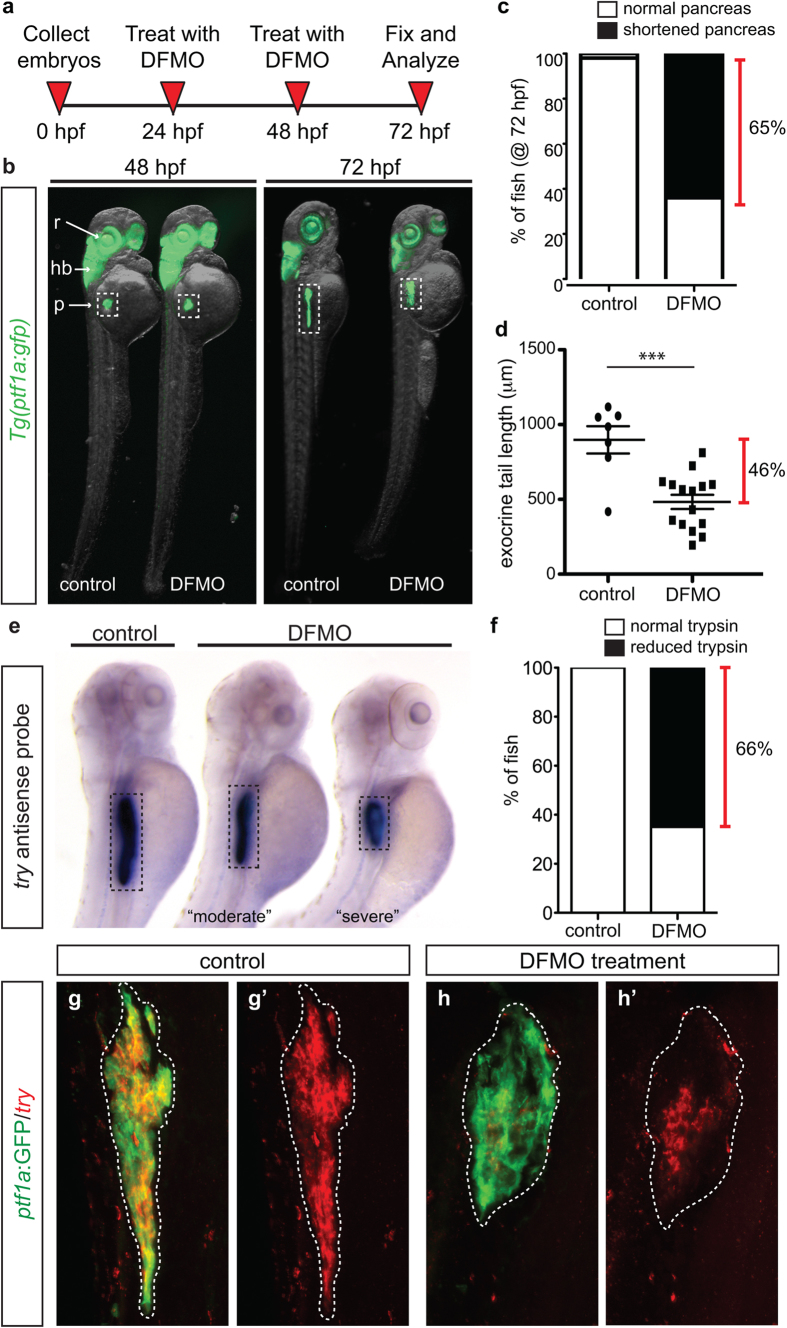
Inhibition of polyamine biosynthesis alters exocrine pancreas development. (**a**) Drug treatment strategy. (**b**) Whole body and exocrine pancreas phenotype in control and DFMO-treated *Tg(ptf1a:gfp)* embryos at 48 hpf and 72 hpf. (**c**) The number of fish at 72 hpf with a normal (n = 30) or a shortened (n = 45) exocrine pancreas, as observed by expression of *ptf1a*:GFP. (**d**) Quantification of exocrine pancreas length in control (n = 7) and DFMO-treated (n = 16) *Tg(ptf1a:gfp)* embryos at 72 hpf (*p* = 0.0002); this represents a 46% difference in exocrine pancreas length. (**e**) Representative images from *in situ* hybridization for expression of the gene encoding trypsin (*try*) on control and DFMO-treated embryos at 72 hpf. (**f**) Number of control (n = 24) and DFMO-treated (n = 24) embryos with a normal or reduced trypsin (*try*) expression domain at 72 hpf. Wholemount fluorescent *in situ* hybridization for trypsin gene expression (*try*) was performed on (**g**) control and (**h**) DFMO-treated *Tg(ptf1a:gfp)* embryos. The corresponding maximum intensity projection of *try* expression in (**g’**) control and (**h’**) DFMO-treated *Tg(ptf1a:gfp)* embryos; the white dotted line outlines the *ptf1a:*GFP + exocrine pancreas domain. r, retina; hb, hindbrain; p, pancreas.

In zebrafish, as in mammals, pancreas development begins with the specification of two separate pancreatic buds that merge to become one organ. The progenitor cell field comprising the dorsal pancreatic bud specifies only endocrine cells that nucleate the principal islet, with the initial wave of endocrine cells clustering by 24 hpf; the progenitors comprising the ventral bud differentiate (after 24 hpf) into both endocrine and exocrine cells^23^. The transcription factor Ptf1a marks early pancreatic progenitor cells and later differentiated acinar cells, as well as cells in the retina and hindbrain^3^,^24^,^25^; therefore *Tg(ptf1a:gfp)* transgenic zebrafish embryos were used to determine the effect of DFMO treatment on exocrine pancreas development. As outlined in Fig. 1a, *Tg(ptf1a:gfp)* embryos were collected from in cross matings and permitted to develop until 24 hours post fertilization (hpf). At 24 hpf, embryos were bathed in egg water containing DFMO. Because ODC is expected to have pleiotropic roles throughout development, a dose curve was performed to determine the effective dose of 1% w/v DFMO that resulted in endodermal phenotypes, yet produced minimal death or body truncation at 48 and 72 hpf (Supplementary Fig. S2a–c). At 48 hpf, *ptf1a*:GFP expressing organs were identical between control and DFMO-treated embryos; however at 72 hpf, the pancreas was reduced in size (Fig. 1b; Supplementary Fig. S2d). An overtly shortened pancreas was apparent in 65% of DFMO-treated embryos (Fig. 1c), and measurement of the length of the *ptf1a:*GFP + pancreatic domain revealed that the exocrine pancreas of DFMO-treated embryos was 46% shorter than that of control embryos (Fig. 1d).

To determine if exocrine differentiation occurred in the truncated *ptf1a*:GFP + pancreatic domain, whole mount *in situ* hybridization (ISH) was performed to assess expression of the trypsin gene (*try*), which is expressed in differentiated exocrine cells. Compared with controls, DFMO-treated embryos at 72 hpf displayed either a moderate or severe reduction in the trypsin domain, with an uneven or patchy pattern of expression (Fig. 1e). In total, 66% of DFMO-treated embryos displayed this reduced trypsin expression domain (Fig. 1f), which is nearly identical to the percentage of DFMO-treated embryos with phenotypic shortened pancreas (Fig. 1c). Subsequent use of fluorescent whole mount ISH permitted analysis of the specific overlap between the *ptf1a*:GFP exocrine domain and the differentiated trypsin-expressing cells. Whereas control pancreas (72 hpf) showed complete overlap between *ptf1a*:GFP and *try* (Fig. 1g), only a portion of the shortened *ptf1a*:GFP pancreas domain expressed *try* after DFMO treatment (Fig. 1h). These data reveal that blocking polyamine biosynthesis by pharmacological inhibition of ODC blunts the differentiation of acinar cells and impedes exocrine pancreas growth.

### Exocrine differentiation but not progenitor cell specification is altered by inhibition of polyamine biosynthesis

**Figure 2:**
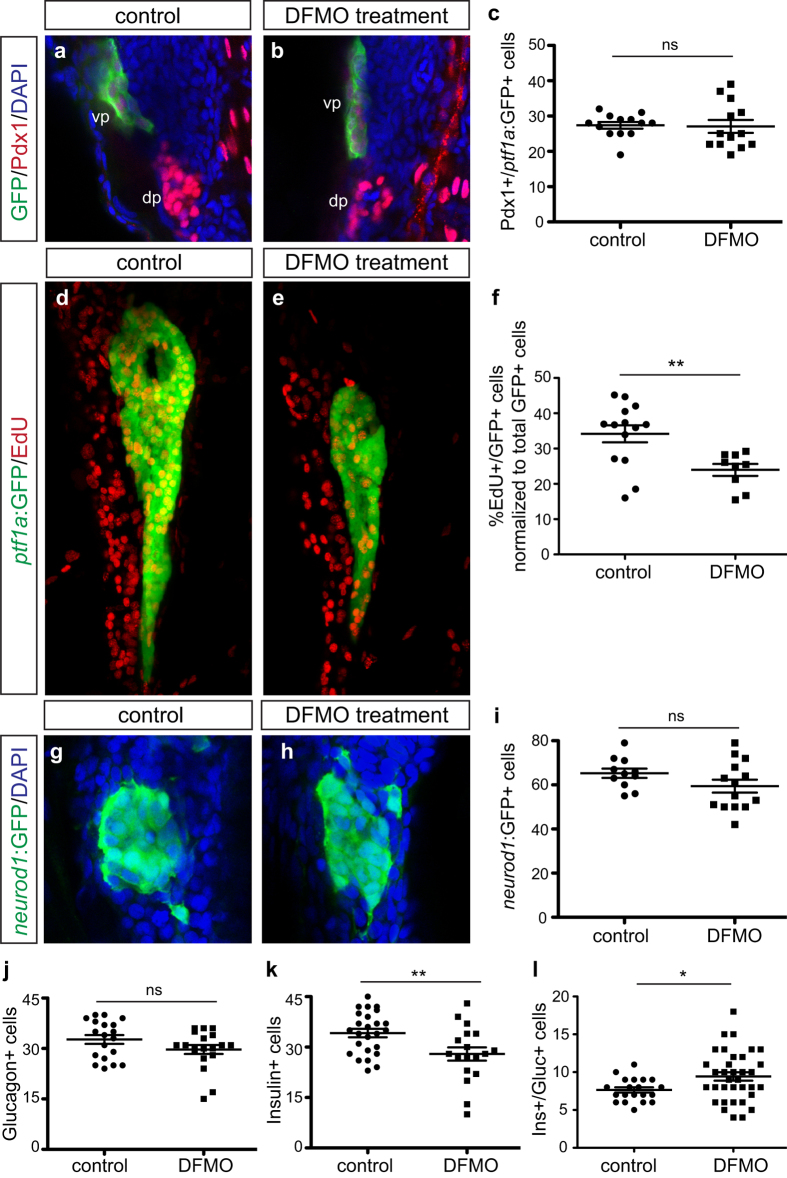
Exocrine and endocrine cell differentiation but not specification is disrupted by DFMO. Representative images of (**a**) control and (**b**) DFMO-treated *Tg(ptf1a:gfp)* embryos stained for Pdx1 and *ptf1a*:GFP to identify co-expressing ventral pancreas progenitor cells. (**c**) Quantification of Pdx1+/ *ptf1a* :GFP + progenitor cells in control (n = 13) and DFMO-treated (n = 13) embryos at 33 hpf (*p* = 0.8823). Representative images of pancreas from (**d**) control and (**e**) DFMO-treated *Tg(ptf1a:gfp)* embryos at 72 hpf after EdU incorporation. (**f**) Quantification of the number of EdU-expressing *ptf1a:*GFP + exocrine cells, normalized to total GFP + cells, in control (n = 14) and DFMO-treated (n = 9) embryos (*p* = 0.0055). Representative images of *neurod1*:GFP + endocrine (islet) cells in (**g**) control and (**h**) DFMO-treated *Tg(neurod1:gfp)* embryos at 72 hpf. (**i**) Quantitation of the *neurod1*:GFP + endocrine cell population at 72 hpf in control (n = 11) and DFMO-treated (n = 14) embryos (*p* = 0.1392). Specific endocrine cell populations were also quantified, including (**j**) Glucagon + α cells (n = 19 for both groups; *p* = 0.1168), (**k**) Insulin + β cells (control n = 25; DFMO n = 18; *p* = 0.0079), and (**l**) Insulin+/Glucagon + co-expressing cells (control n = 20; DFMO n = 35; *p* = 0.0282). *p < 0.05, **p < 0.01, ***p < 0.001; ns, not significant; dp, dorsal pancreas; vp, ventral pancreas.

A reduction in overall exocrine pancreas size could be attributable to a decrease in the total number of progenitor cells specified in the early pancreatic domain, or impaired proliferative expansion of the acinar cells that do differentiate from these progenitors. Thus, to determine if polyamine depletion altered the initial pancreas progenitor cell pool, *Tg(ptf1a:gfp)* embryos were treated with DFMO from 24 hpf to 33 hpf. This stage (33 hpf) was chosen for analysis because a substantial population of undifferentiated ventral pancreas progenitors is present; this population of cells is similar to the Cpa1 + multipotent progenitor cells identified in mouse, which give rise to differentiated acinar cells^8^,^26^. Pancreas progenitor cells co-expressing Pdx1 and *ptf1a*:GFP were quantitated in control and DFMO-treated embryos at 33 hpf (Fig. 2a,b); this analysis determined that the progenitor cell population was unaltered between control and DFMO-treated embryos (Fig. 2c). Therefore the ventral pancreas progenitor cell pool is not altered by inhibition of polyamine biosynthesis.

We next evaluated EdU incorporation into exocrine cells at 72 hpf to determine if the block in exocrine differentiation was associated with altered cellular proliferation. Whereas EdU incorporation was apparent in both control and DFMO-treated embryos (Fig. 2d,e), the number of EdU+/*ptf1a*:GFP + cells was significantly reduced in the latter (Fig. 2f). Therefore, among the population of differentiating exocrine cells there is a reduction in proliferation. Together these data indicate strongly that a decrease in both differentiation and proliferation contributes to a significant reduction in exocrine pancreas size.

### Polyamine depletion alters β cell differentiation but not total endocrine cell specification

The zebrafish pancreas, like that of mouse and human, is composed of exocrine cells as well as hormone-producing endocrine cells, including glucagon-producing α cells and insulin-producing β cells. Given that polyamine levels are elevated in both exocrine and endocrine cells in the pancreas^16^,^17^, we hypothesized that endocrine development would also be affected following inhibition of polyamine biosynthesis. In zebrafish, all nascent endocrine cells express the transcription factor neuronal differentiation 1 (*neurod1*) prior to terminal differentiation and hormone expression^27^. Thus, to investigate the endocrine population we utilized the *Tg(neurod1:gfp)* transgenic line. *Tg(neurod1:gfp)* embryos were treated with DFMO as described above, and total endocrine cell number (*neurod1*:GFP + cells) (Fig. 2g,h), as well as individual populations of α and β cells, was quantitated. Whereas the *neurod1*:GFP + total endocrine cell population (Fig. 2i) and glucagon + α cell population (Fig. 2j; Supplementary Fig. S3a,b) were unchanged in DFMO-treated embryos at 72 hpf, the insulin + β cells were reduced in number when compared with controls (Fig. 2k; Supplementary Fig. S3c,d). DFMO treatment of double transgenic zebrafish embryos (*Tg(gcga:gfp);Tg(ins:dsRed*)) permitted the subsequent analysis of the insulin/glucagon co-expressing cells. Interestingly, the number of insulin/glucagon co-expressing cells was significantly increased in DFMO-treated embryos compared with controls (Fig. 2l; Supplementary Fig. S3e,f); however, alterations in β cell number did not impact overall free glucose levels in DFMO-treated embryos as compared with controls (Supplementary Fig. S3g). Taken together these results signify that, similar to the pancreas progenitor cells, total endocrine cell number is unaltered when polyamine biosynthesis is interrupted; however, the subsequent differentiation of insulin-producing β cells is sensitive to altered polyamine biosynthesis. Moreover, the increase in the number of insulin/glucagon co-expressing cells together with the reduction in total β cell number suggests that inhibition of polyamine biosynthesis impedes the maturation of β cells.

**Figure 3:**
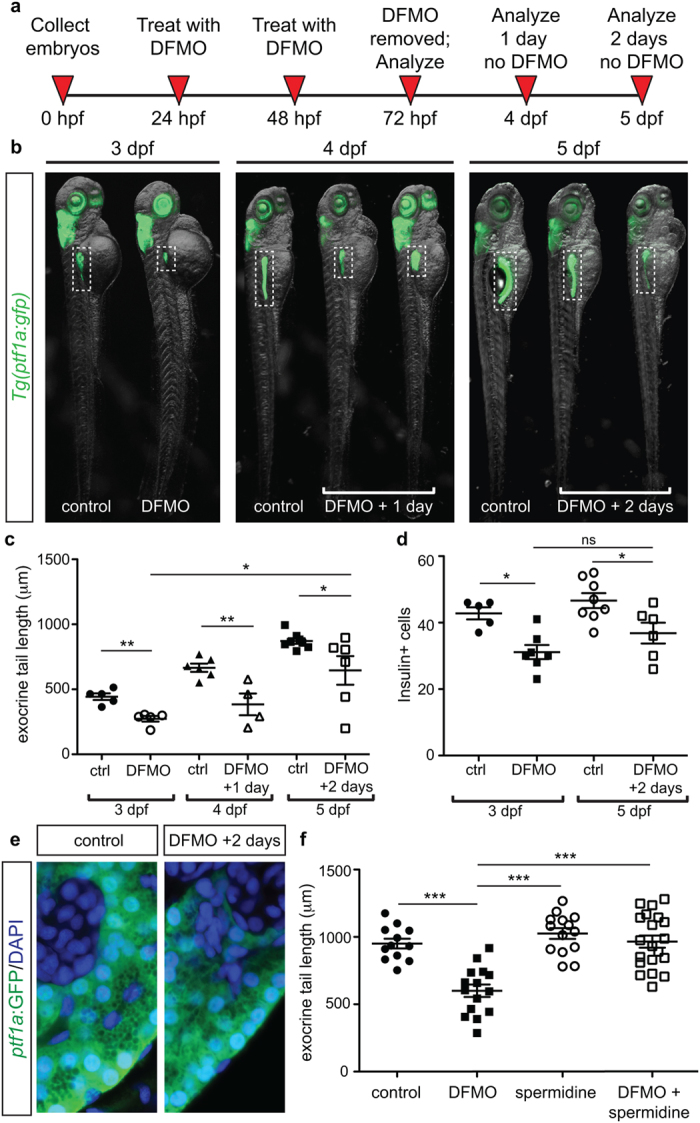
Withdrawal of DFMO treatment causes partial recovery, and spermidine supplementation rescues phenotypic defects. (**a**) Strategy for DFMO treatment and withdrawal. (**b**) Representative images of control and DFMO-treated *Tg(ptf1a:gfp)* embryos after 0, 1, or 2 days of DFMO treatment withdrawal; white dotted box denotes the location of the pancreas (**c**) Quantification of exocrine pancreas length in control (n = 5) and DFMO-treated (n = 5) *Tg(ptf1a:gfp)* embryos at 3 dpf (*p* = 0.001); control (n = 6) and DFMO-treated (n = 4) *Tg(ptf1a:gfp)* embryos at 4 dpf (one day after DFMO was removed)(*p* = 0.0064); control (n = 7) and DFMO-treated (n = 6) *Tg(ptf1a:gfp)* embryos at 5 dpf (two days after DFMO was removed)(*p* = 0.0381); a significant increase in exocrine pancreas length was observed between embryos that were DFMO-treated compared with DFMO + 2 day withdrawal (*p* = 0.0226). (**d**) The insulin + β cell population was also quantified for 3 dpf control (n = 5) and DFMO-treated embryos (n = 7) (*p* < 0.05), as well as 5 dpf control (n = 8) and DFMO + 2 day withdrawal embryos (n = 6) (*p* < 0.05); no significant difference in β cell number was observed after 2 days of DFMO withdrawal (*p* = 0.1512). (**e**) Representative images of *ptf1a:*GFP + exocrine pancreas cells from control and DFMO + 2 day withdrawal (5 dpf) *Tg(ptf1a:gfp)* embryos, illustrating the presence of zymogen granules in the *ptf1a:*GFP + cells. (**f**) Quantification of exocrine pancreas length in control (n = 12), DFMO-treated (n = 15), spermidine-treated (n = 14), and DFMO  + spermidine-treated (n = 20) *Tg(ptf1a:gfp)* embryos at 72 hpf. Whereas DFMO treatment resulted in reduced exocrine pancreas length (*p* < 0.0001), the exocrine pancreas in embryos treated with spermidine alone was longer than DFMO-treated (*p* < 0.0001) but no different from wildtype (*p* = 0.1843). DFMO-treatment supplemented with spermidine showed exocrine pancreas length no different from wildtype (*p* = 0.8176) and significantly longer compared with DFMO-treated embryos (*p* < 0.0001). dpf, days post fertilization; *p < 0.05, **p < 0.01, ***p < 0.001; ns, not significant.

To determine if any of the pancreatic cell alterations that resulted from pharmacological inhibition of ODC were reversible, *Tg(ptf1a:gfp)* embryos were treated with DFMO from 24 hpf to 72 hpf, then washed and recovered in fresh egg water for either 24 or 48 hours (Fig. 3a). Quantification identified that at 4 dpf and 5 dpf there was a persistent decrease in exocrine pancreas length, as compared with age-matched controls (Fig. 3b,c). However, the exocrine pancreas length in embryos that were permitted to recover for 2 days (5 dpf) was significantly longer than DFMO-treated embryos at 3 dpf (Fig. 3b,c). In contrast, there was no recovery of insulin + β cell number (Fig. 3d). These data suggest that the effects of inhibition of polyamine biosynthesis on exocrine, but not endocrine, pancreas development are reversible. In fact, careful examination of the cytoplasmic compartment of *ptf1a*:GFP + exocrine cells following two days of DFMO withdrawal (5 dpf) identified the presence of zymogen granules, which are characteristic of properly differentiated exocrine cells (Fig. 3e).

### Spermidine rescues the phenotypic defects caused by ODC inhibition

In light of our finding that polyamine biosynthesis (via Odc1) is essential for pancreatic cell development, we next tested whether a specific downstream pathway mediates this requirement. Spermidine is one of the polyamines synthesized downstream of the reaction catalyzed by ODC. To determine whether polyamine supplementation could prevent the exocrine pancreas phenotype, we treated *Tg(ptf1a:gfp)* embryos simultaneously with DFMO and spermidine at 24 hpf and analyzed the effects at 72 hpf. A dose titration was performed to determine that a dose of 1 mM spermidine produced minimal death or body truncation (data not shown). Whereas spermidine alone had no effect on exocrine pancreas length, the combined treatment of DFMO and spermidine resulted in a complete rescue of the exocrine pancreas phenotype to wildtype length (Fig. 3f). These data suggest that restoration of polyamine levels through the addition of spermidine can bypass the inhibitory effect of DFMO on pancreatic cell development.

**Figure 4:**
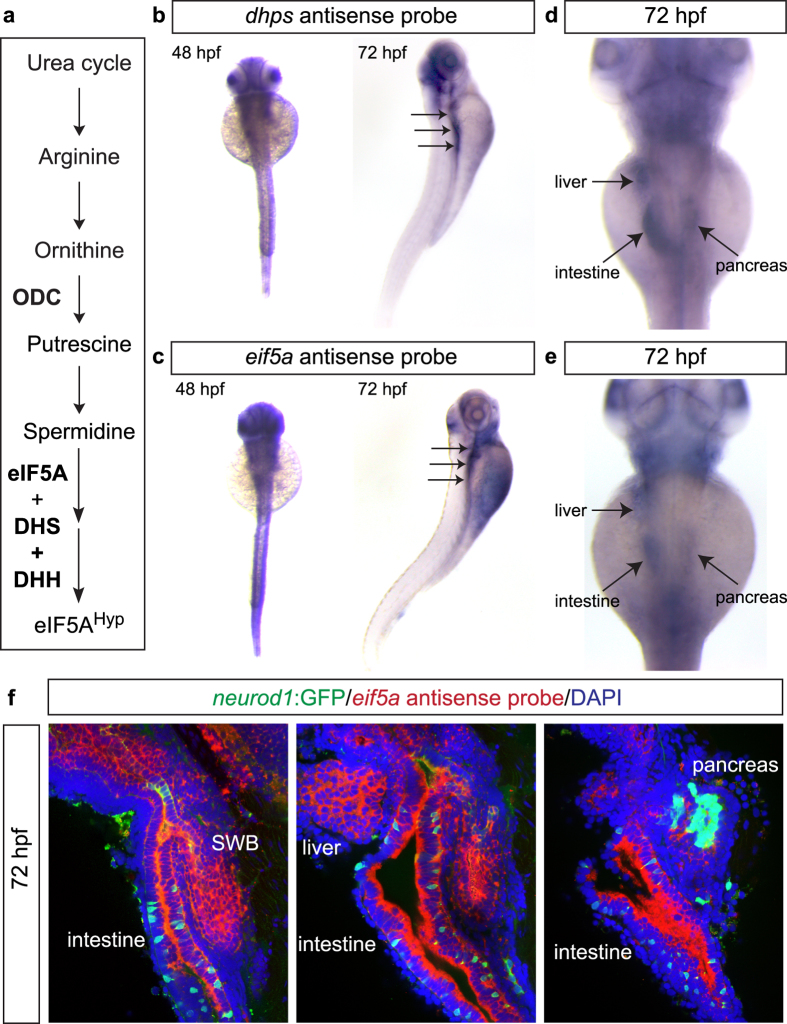
Expression patterns of *dhps* and *eif5a* in zebrafish. (**a**) Pathway illustrating the connection between the polyamine biosynthetic pathway, eIF5A, and DHS. Wholemount *in situ* hybridization for (**b**) *dhps* and (**c**) *eif5a* at 48 hpf and 72 hpf. At 72 hpf, higher magnification reveals expression of (**d**) *dhps* and (**e**) *eif5a* in the liver, intestine and pancreas. (**f**) Fluorescent *in situ* hybridization of *eif5a* in *Tg(neurod1:gfp)* transgenic fish at 72 hpf confirms expression in endoderm-derived organs, including swim bladder (SWB), intestine, liver, and pancreas.

One known role of the polyamine spermidine is that it serves as a substrate for the production of the active form of the eukaryotic mRNA translation factor, eIF5A. The posttranslational modification of eIF5A, known as “hypusination”, requires spermidine, the enzymes deoxyhypusine synthase (DHS) and deoxyhypusine hydroxylase (DHH), and the Lys50 residue of eIF5A (Fig. 4a). Importantly, DHS is the rate limiting enzyme catalyzing the addition of hypusine [N(ε) (4-amino-2-hydroxybutyl)lysine] to eIF5A (eIF5A^Hyp^)^28^,^29^,^30^. This process, and its connection to polyamine biosynthesis, has never been studied in the context pancreas development. Interestingly, western blot analysis of control and DFMO-treated embryos identified no difference in the expression of DHS, but a significant reduction in the expression of the hypusinated form of eIF5A (eIF5A^Hyp^)(Supplementary Figure S3h–j), which points to a direct connection between polyamine biosynthesis and the downstream process of mRNA translation involving eIF5A^Hyp^.

### Knockdown of ***dhps*** by morpholino phenocopies inhibition of polyamine biosynthesis

To further investigate this connection between polyamine biosynthesis and eIF5A^Hyp^-mediated mRNA translation, we first examined the embryonic expression of *dhps* and *eif5a* and then used a knockdown approach to examine pancreas developmental phenotypes in the absence of DHS, the rate-limiting enzyme in the process of hypusination. Similar to Odc1, protein sequence alignment (ClustalW^22^) revealed that zebrafish Dhps and Eif5a are 76% and 82% identical, respectively, to their mouse and human orthologs (Supplementary Fig. S4). Whole mount ISH analysis identified a similar expression pattern between *dhps* and *eif5a* in zebrafish embryos (Fig. 4b,c). Moreover, this expression pattern was similar to that of *odc1* (data from the Zebrafish Information Network (ZFIN), University of Oregon, Eugene, OR 97403-5274; http://zfin.org/; 12/3/2014). Of particular interest, expression was observed in endoderm-derived organs, including the liver, intestine and pancreas (Fig. 4d,e). Higher resolution fluorescent ISH confirmed localization to the endodermal organs at 72 hpf (Fig. 4f). One day earlier (48 hpf), expression in the pancreas was also identified, with expression observed in both the exocrine and endocrine compartments (endocrine visualized by expression of *neurod1*:GFP) (Supplementary Fig. S5).

**Figure 5:**
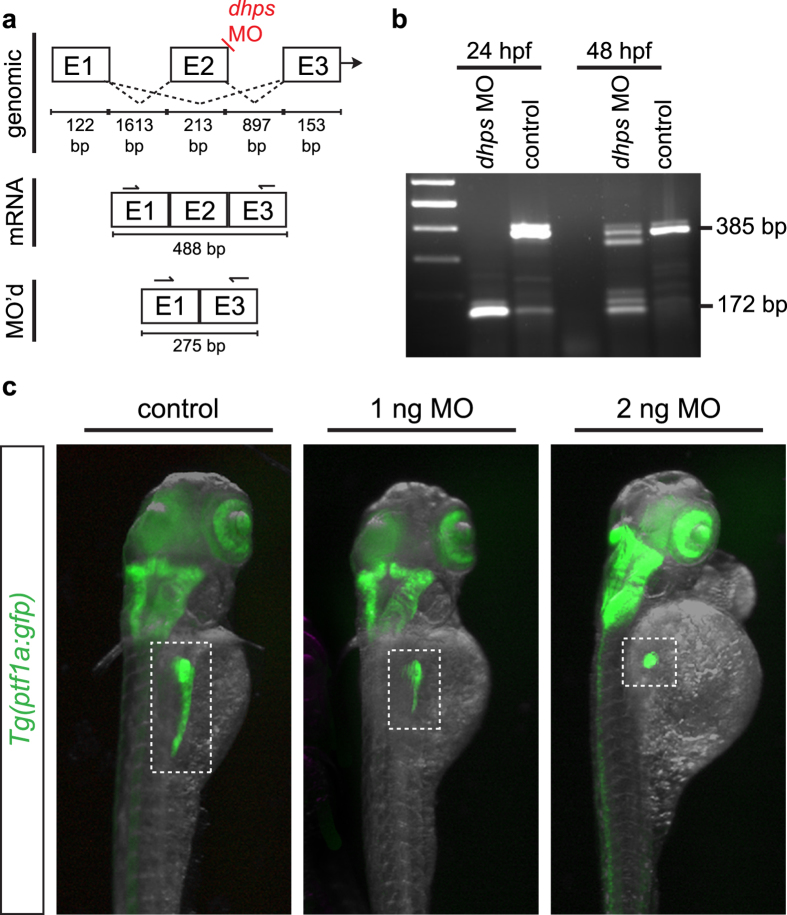
Knockdown of *dhps* in the developing zebrafish. (**a**) Design of the *dhps* splice-blocking morpholino (MO). (**b**) Injection of the *dhps* MO results in a loss of exon 2, as confirmed by RT-PCR from control and MO-injected *Tg(ptf1a:gfp)* embryos; wildtype *dhps*, 385 bp; E2 deletion in *dhps*, 172 bp. (**c**) *dhps* MO-injected *Tg(ptf1a:gfp)* embryos at 72 hpf show a significant pancreatic phenotype after low dose MO injection (1 ng or 2 ng). E, exon; bp, base pair.

With pancreatic expression of *dhps* and *eif5a* confirmed, we next tested the hypothesis that DHS lies functionally downstream of ODC by generating a splice-blocking morpholino (MO) to knockdown *dhps* in the developing zebrafish embryo. By design, the MO interrupted splicing of exons 2 and 3, and caused a deletion of exon 2 in zygotically transcribed *dhps* (Fig. 5a). A dose curve was performed to determine a partial yet effective *dhps* knockdown; zygotic injection of 1 ng or 2 ng of *dhps* MO produced an altered phenotype in the pancreas of *Tg(ptf1a:gfp)* larvae at 72 hpf without significant death or disruption to overall embryonic development (Supplementary Fig. S6a,b). To confirm efficacy of the *dhps* MO, RT-PCR was performed from control and MO-injected embryos, and subsequent sequencing of the PCR product confirmed deletion of exon 2 (Fig. 5b; Supplementary Fig. S6c). Although no overt changes were observed in the *ptf1a*:GFP-expressing retina and hindbrain, the increasing *dhps* MO doses resulted in increasing severity of a truncated pancreas phenotype (Fig. 5c).

**Figure 6:**
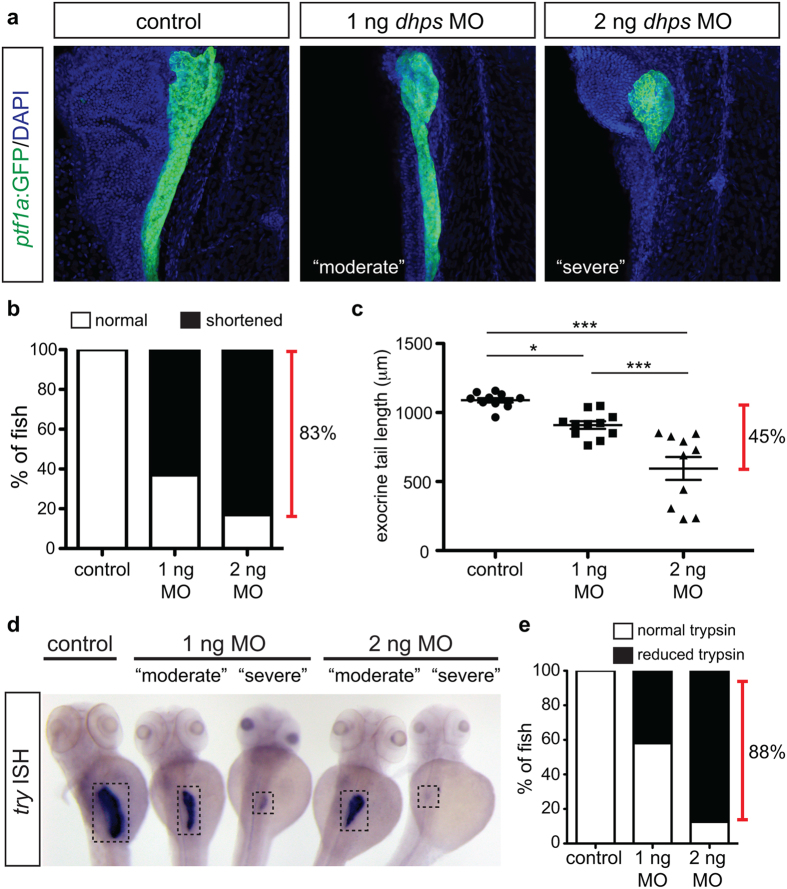
Knockdown of *dhps* inhibits exocrine cell differentiation. (**a**) Representative images of the pancreas from control, 1 ng MO-injected, and 2 ng MO-injected *Tg(ptf1a:gfp)* embryos; images also depict characteristic pancreata with a “moderate” or “severe” defect in exocrine length. (**b**) The percentage of control (n = 20), 1 ng MO-injected (n = 46), and 2 ng MO-injected (n = 23) embryos with a normal or shortened exocrine pancreas at 72 hpf, as observed by expression of *ptf1a:*GFP. (**c**) Quantification of exocrine pancreas length in control (n = 11), 1 ng MO-injected (n = 11), and 2 ng MO-injected (n = 10) embryos (*p* < 0.0001) at 72 hpf; this shows a 45% difference in exocrine pancreas length. (**d**) Representative images of *in situ* hybridization for *try* (gene encoding trypsin) on control and MO-injected embryos at 72 hpf. (**e**) The percentage of control (n = 15), 1 ng MO-injected (n = 5), and 2 ng MO-injected (n = 19) embryos with normal or reduced expression of trypsin at 72 hpf. MO, morpholino; *p < 0.05, **p < 0.01, ***p < 0.001.

Similar to the phenotype observed in DFMO-treated embryos, knockdown of *dhps* impaired exocrine pancreas development. The majority of *dhps* MO-injected embryos displayed a shortened exocrine pancreas (Fig. 6a,b). Measurement of the *ptf1a*:GFP + exocrine pancreas domain identified a significant reduction in pancreas length between control and either 1 ng or 2 ng *dhps* MO-injected embryos; 2 ng *dhps* MO-injected embryos showed the greatest size reduction with a 45% difference in length compared with controls (Fig. 6c). This truncated pancreas phenotype is nearly identical to that observed after DFMO-induced inhibition of polyamine biosynthesis (Fig. 1d). To examine exocrine cell differentiation in the *ptf1a*:GFP + pancreatic remnant of *dhps* MO-injected embryos, whole mount ISH was performed for *try* (Fig. 6d); 42% and 88% of 1 ng and 2 ng *dhps* MO-injected embryos, respectively, displayed a reduced trypsin expression domain at 72 hpf (Fig. 6e). Taken together these data reveal that knockdown of *dhps* closely phenocopies inhibition of ODC, and likely places the action of DHS in the same pathway as ODC.

### Knockdown of ***dhps*** inhibits β cell differentiation

**Figure 7:**
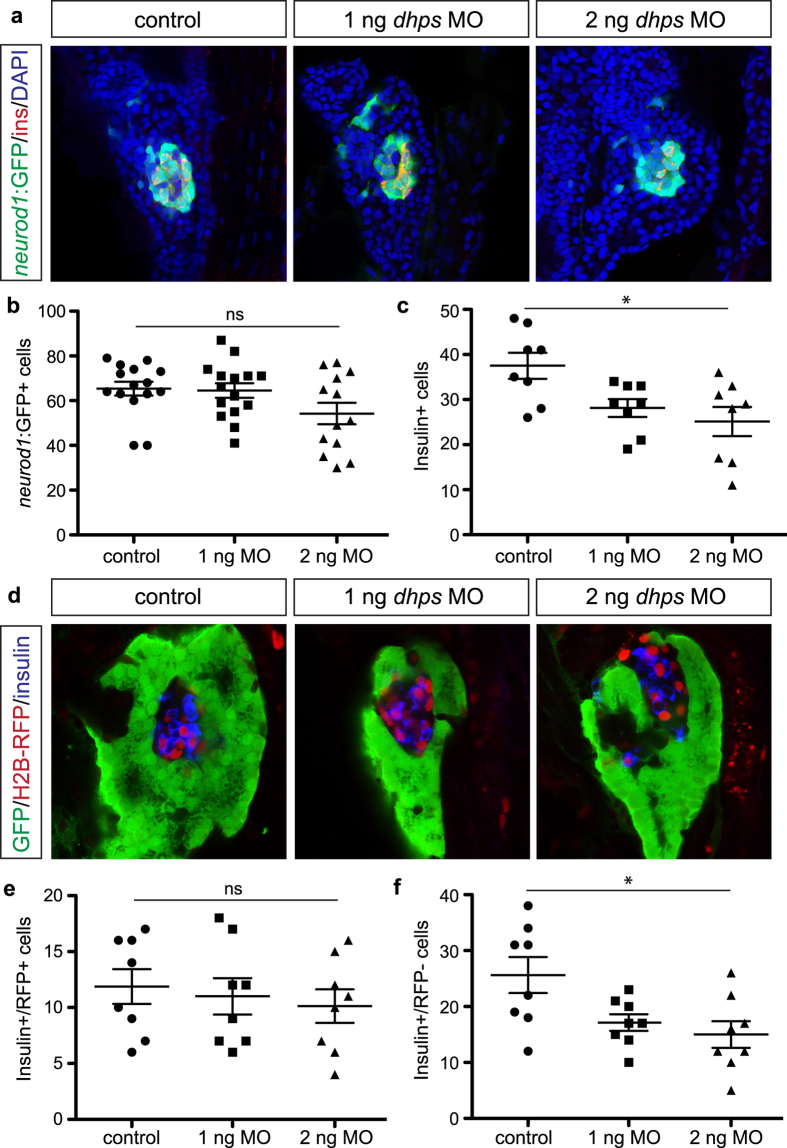
Knockdown of *dhps* inhibits β cell differentiation. (**a**) Representative images of *neurod1*:GFP + endocrine cells in the pancreas of control, 1 ng MO-injected, and 2 ng MO-injected *Tg(neurod1:gfp)* embryos at 72 hpf. Quantification of the number of (**b**) *neurod1*:GFP + cells in control (n = 15), 1 ng MO-injected (n = 15), and 2 ng MO-injected (n = 13) embryos (*p* = 0.0783), and (**c**) insulin + β cells in control (n = 8), 1 ng MO-injected (n = 8), and 2 ng MO-injected (n = 8) embryos (*p* = 0.012), at 72 hpf. (**d**) Representative images of the insulin + β cells from control, 1 ng MO-injected, and 2 ng MO-injected *Tg(ptf1a:gfp)* embryos at 72 hpf after label-retaining cell (LRC) assay was performed. LRC + cells express H2B-RFP. Quantification of the number of (**e**) insulin+/RFP + cells (LRC + or dorsal pancreas-derived cells) in control (n = 8), 1 ng MO-injected (n = 8), and 2 ng MO-injected (n = 8) embryos (*p* = 0.734), and (**f**) insulin+/RFP- cells (LRC- or ventral pancreas-derived cells) in control (n = 8), 1 ng MO-injected (n = 8), and 2 ng MO-injected (n = 8) embryos (*p* = 0.0147), at 72 hpf. *p < 0.05, **p < 0.01, ***p < 0.001; ns, not significant.

To quantitate the total endocrine cell population after knockdown of *dhps*, we utilized *Tg(neurod1:gfp)* transgenic zebrafish embryos. As shown in Fig. 7a,b, *dhps* MO-injected embryos displayed no change in the total number of *neurod1:*GFP + endocrine cells. However, similar to DFMO-treated embryos, a significant reduction in the insulin + β cell population was observed (Fig. 7c). Using the zebrafish embryo made it possible to also determine the origin of the β cells that were altered with loss of *dhps* by performing a label retaining cell (LRC) assay^31^,^32^. This assay uses H2B-RFP mRNA simultaneously co-injected with the *dhps* MO to uniformly label all early embryonic cells; as development proceeds, quiescent cells retain RFP expression (including dorsal pancreas-derived cells) while dividing cells (including ventral pancreas-derived cells) dilute the RFP signal (Fig. 7d). The LRC assay indicated that β cells derived earlier, from dorsal pancreatic progenitor cells (RFP+), were unaltered in number (Fig. 7e) whereas those derived later, from the ventral pancreatic progenitor cells (RFP-), were significantly reduced in number (Fig. 7f).

**Figure 8:**
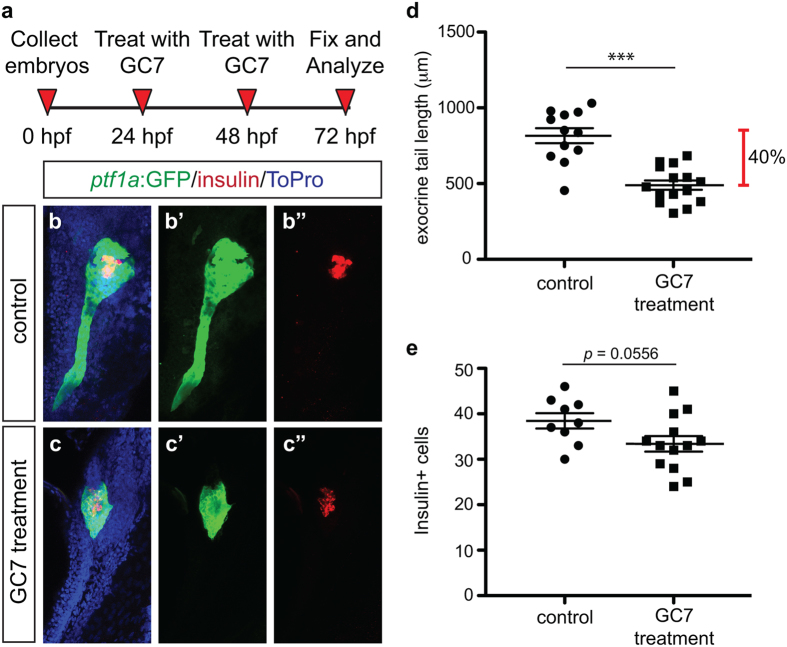
Pharmacological inhibition of DHS with GC7 phenocopies genetic loss of *dhps* and inhibition of ODC. (**a**) Inhibitor treatment strategy. Representative maximum intensity projection images of the pancreas from (**b**) control and (**c**) GC7-treated *Tg(ptf1a:gfp)* embryos; for comparison single channel images are also provided for *ptf1a*:GFP expression in (**b’**) control and (**c’**) GC7-treated embryos, and insulin expression in (**b”**) control and (**c”**) GC7-treated embryos. (**d**) Quantification of exocrine pancreas length in control (n = 12) and GC7-treated (n = 15) *Tg(ptf1a:gfp)* embryos at 72 hpf (*p* < 0.0001); this represents a 40% difference in exocrine pancreas length. (**e**) Quantification of insulin + β cell number in control (n = 9) and GC7-treated (n = 13) *Tg(ptf1a:gfp)* embryos at 72 hpf (*p* = 0.0556). *p < 0.05, **p < 0.01, ***p < 0.001.

To confirm that loss of DHS alters both exocrine cell and β cell development, *Tg(ptf1a:gfp)* embryos were treated with the DHS inhibitor N1-guanyl-1,7-diaminoheptane (GC7)^33^ using an experimental design identical to that of treatment with DFMO (Fig. 8a). A dose titration was performed to determine that a dose of 20mM GC7 resulted in endodermal phenotypes, yet produced minimal death or body truncation (data not shown). GC7 treated embryos were analyzed at 72 hpf and displayed a reduced *ptf1a*:GFP + exocrine pancreas domain (Fig. 8b–d), as well as a reduction in the number of insulin + cells (Fig. 8b,c,e). These analyses confirm that knockdown of *dhps* or functional inhibition of DHS, similar to inhibition of polyamine biosynthesis by DFMO, results in a reduction of exocrine pancreas and β cell mass.

## Discussion

Whereas polyamine biosynthesis is known to be important for cellular proliferation and essential for the earliest stages of embryonic development, this pathway has never been studied in the context of organ development. The zebrafish has many characteristics that make it an ideal model for studying vertebrate organogenesis and cell fate determination, including optical transparency, external fertilization, facile genetic manipulation, and rapid development. Given that pancreatic organogenesis in zebrafish closely approximates the mammalian process, we utilized this model system and, in particular, numerous pancreas cell-specific transgenic reporter strains to dissect the function of polyamine biosynthesis in pancreatic organogenesis.

Our study determined that inhibiting polyamine biosynthesis does not alter pancreas progenitor (Pdx1+/Ptf1a+) cell number; however, the dramatic phenotype observed of a shortened exocrine pancreas after treatment with DFMO or knockdown/inhibition of DHS defines a role for polyamines in both the proliferation and differentiation of exocrine cells. This expanded function of polyamines in the pancreas may permit reinterpretation of previous studies. In particular, DFMO treatment of pancreas after chemically-induced (caerulein) injury only implicated altered proliferation as the cause of the reduced exocrine pancreas regrowth/regeneration^34^,^35^. The results of our study would suggest that DFMO treatment altered proliferation as well as the differentiation of exocrine cells from injury-stimulated progenitors^36^,^37^,^38^,^39^,^40^,^41^. Likewise, genetic models displaying reduced exocrine differentiation, such as the pancreas-specific deletion of *c-myc*^42^, could possibly be rescued by induction of polyamine biosynthesis.

The embryonic pancreas has a known population of multihormonal insulin/glucagon co-expressing endocrine cells. Early lineage tracing studies in mouse refuted the notion that these cells are β cell progenitors^43^; however, a decade of research and advancements in technology have rekindled the debate. Based on more recent evidence in the mouse, it has been proposed that insulin/glucagon co-expressing cells reflect a population of cells expressing proglucagon that may serve as β cell precursors (a “pro-α” cell population)^44^. Likewise evidence from zebrafish points to these multihormonal cells as an immature endocrine cell population during development with the potential to give rise to either α or β cells^45^. In our model, inhibition of polyamine biosynthesis does not alter α cell number but does reduce the β cell population and concomitantly increase the number of insulin/glucagon co-expressing cells. These data suggest that even when polyamine biosynthesis is inhibited, immature endocrine cells continue differentiating toward the α cell lineage, but that additional signals including increased cellular polyamines are required to drive differentiation toward the β cell lineage. This is in concordance with previous studies that showed exogenous polyamine supplementation could promote insulin expression^46^, as well as neogenesis of β cells from duct-associated progenitors in the pancreata of rats injured by alloxan treatment^47^. Together, these findings further support the idea that polyamines in the pancreas have an alternate cellular function than simply proliferation—these aliphatic amines also function in the process of cellular differentiation.

Loss of *dhps* results in embryonic lethality in mice^48^. Our study was designed to investigate progressive stages of pancreas development—from the progenitor cell stage through to differentiated exocrine and endocrine cells. Moreover, our use of partial knockdowns via morpholino and small molecule inhibitors permitted the evaluation of specific aspects of pancreas development at specific stages. We used a splice-blocking morpholino with the deliberate intent of generating a delayed knockdown by only disrupting zygotic zebrafish *dhps*. We exploited this aspect of splice-blocking morpholino design to permit normal development in the earliest embryonic stages, due to maternally contributed *dhps* expression, but knockdown of *dhps* during later organogenesis; the resulting experimental embryos had clear disruption of *dhps* (Fig. 5; Supplementary Fig. S6). In addition, the combined use of morpholino and LRC assay allowed for the evaluation of differentiated endocrine cells derived from two discrete progenitor sources. In zebrafish, dorsal pancreas-derived endocrine cells are similar to the “first wave endocrine cells” described in the mouse^49^ whereas ventral pancreas derived endocrine cells are akin to those that differentiate during the secondary transition in mouse^50^. Moreover, there is evidence that these two pools of endocrine cells may be functionally different^31^. Our use of the LRC assay permitted these populations to be segregated and evaluated, ultimately determining that inhibition of polyamine biosynthesis alters the β cells derived from the later specified ventral pancreas progenitors, which appear to be the population of endocrine cells of functional significance to carbohydrate metabolism.

The reduction in β cell number as a result of ODC or DHS inhibition also raises the question of whether there is an overall effect on glucose metabolism in embryos where polyamine biosynthesis is inhibited. In the mouse, altered glucose homeostasis is observed with near complete β cell loss (e.g. high dose streptozotocin treatment), or disruption to the normal function of organs important in maintaining whole body metabolism (e.g. liver, brain). Regrettably many routine analyses in mouse cannot be performed in zebrafish embryos; however, we were able to evaluate whole embryo free glucose levels as a surrogate measure of metabolic health. Our data demonstrated no change in glucose levels following DFMO treatment, which implies that the reduction in β cell number, although statistically significant, is not large enough to result in altered glucose homeostasis. Whereas it remains possible that other organs important for the maintenance of proper metabolic health are also affected by DFMO treatment, the euglycemia observed in DFMO-treated embryos suggests that inhibition of polyamine biosynthesis does not disrupt normal glucose metabolism.

In the pursuit of expanding our understanding of how polyamine biosynthesis functions during pancreatic organogenesis, we have generated data that support a direct connection between polyamine biosynthesis and the downstream process of mRNA translation. Our study showed that inhibition of polyamine biosynthesis (with DFMO) reduced expression of the hypusinated form of eIF5A, and that the pancreatic phenotype after DFMO treatment was identical to that of *dhps* knockdown (with MO) and DHS inhibition (with GC7). Together, these data constitute strong evidence that these factors function in the same pathway. Connecting polyamine biosynthesis with DHS and eIF5A^Hyp^ implicates cellular polyamines as a requirement for proper mRNA translation. Determining how DHS and eIF5A^Hyp^ function in the translation of specific mRNAs important for cellular differentiation is currently under investigation.

Although this relationship between polyamine biosynthesis, mRNA translation, and cellular differentiation is in part speculative it underscores the conceptual advance arising from this work. Collectively the data presented in this study establishes that polyamine biosynthesis is required for cellular differentiation, and that inhibition of the enzyme ODC or the downstream enzyme DHS negatively impacts the differentiation of specific pancreatic cell types. In short, this connection supports the idea that the differentiation of cellular lineages, and in particular the pancreatic β cell lineage, might be influenced by the exogenous administration of polyamines. Moreover, the simplicity of this particular scheme lends itself to be applied to many currently published *in vitro* differentiation protocols from embryonic stem cells^12^,^13^,^14^,^15^. In fact the results of our study would suggest that the application of exogenous polyamines has the potential to enhance the yield of *in vitro* generated insulin-producing β cells for the treatment of persons with diabetes, and is therefore an avenue of current investigation.

## Methods

### Zebrafish (***Danio rerio***) maintenance and strains

All zebrafish transgenic lines were previously published, and raised in standard laboratory conditions at 28.5 °C. The following transgenic lines were used in the experiments: *Tg(ptf1a:gfp)*^*jh1*^ 51, *Tg(neurod1:gfp)*^52^, *Tg(gcga:GFP)*^*ia1*^ 26*, Tg(ins:dsRed)*^*m1018*^ 53. All lines were maintained and used according to the “Guidelines for Use of Zebrafish in the NIH Intramural Research Program” (approved 03/11/09 and revised 05/08/13; http://oacu.od.nih.gov/ARAC/documents/Zebrafish.pdf). All animal procedures were conducted in accordance with OLAW guidelines and were approved by the Indiana University Institutional Animal Care and Use Committee.

### Pharmacological treatments

Zebrafish embryos were collected and cultured in standard conditions at 28.5 °C in egg water supplemented with (4 mM) 1-phenyl 2-thiourea (PTU). Pharmacological inhibitor, 1% w/v difluoromethylornithine (DFMO) (generous gift from Dr. P. Woster) or 20 mM N1-guanyl-1,7-diaminoheptane (GC7) (ProSpec), was then added to the egg water at 24 hpf, refreshed at 48 hpf, and washed out at 72 hpf. The optimal concentration was determined by dose curve, using GFP expression in *Tg(ptf1a:gfp)* embryos as the phenotypic read out, examining embryos for alterations in proper pancreas development without gross alteration in overall morphology.

For experiments involving spermidine supplementation, embryos were cultured in egg water with 1 mM spermidine (Sigma-Aldrich) either alone or in combination with 1% w/v DFMO. The optimal concentration of 1 mM spermidine was determined by dose curve examining embryos for alterations in overall morphology and viability.

### Glucose measurement

Glucose levels in zebrafish embryos were measured using the glucose assay kit, as per the manufacturers instructions (Biovision)^45^.

### Microinjection and morpholino design

The following antisense morpholino (MO) (Gene Tools, LLC) was injected into 1-cell stage embryos: *dhps* MO (targeting NM_213222), 5′- ACGATCAGTCTGTCACTCACCATCT (1 ng and 2 ng), targeting the splice site at the junction of exon 2 and exon 3. *H2BRFP* mRNA was transcribed with SP6 mMessage machine kit (Invitrogen), and 100 pg was co-injected into zygotes along with *dhps* MO. Efficiency of knockdown was determined by RT-PCR, using primers that amplify across the predicted deletion: 5′- GCGCTGTGAAATGTGAGTGAAAC and 5′- GTTTGACGTGTAGCCCAGGAAT. The resultant PCR amplicon was 385 bp in the control embryos and 172 bp in the *dhps* MO-injected embryos, and was visualized by standard agarose gel electrophoresis.

### *In situ* hybridization and immunofluorescence

Whole mount immunofluorescent staining and cell quantifications were performed as previously described^53^. For immunofluorescence the following antibodies were used: chicken anti-GFP (Aves Labs); guinea pig anti-insulin (Biomeda); mouse anti-glucagon (Sigma); rabbit anti-dsRed (Clontech); guinea pig anti-Pdx1 (gift of Dr. C. Wright). Alexa Fluor-conjugated antibodies were used for visualization (Life Technologies), and DAPI (Invitrogen) was used to identify nuclei. For wholemount *in situ* hybridization, antisense probes were transcribed from templates that were generated by PCR from cDNA using primer sets that amplify *eif5a* (5′-TCCAGATCTTGATTTCGCCAG and 5′-GGAGGTTGAGGTGGGGTAA) or *dhps* (5′-GCCTCTGCCGGACAATCT and 5′-CTGCACTGGAAGGCAATGAA). BM purple (Roche Life Sciences), Vector Red (Vector Laboratories), or Fast Red (Sigma-Aldrich) was used for visualization. For Z-stack images and cell quantification, embryos were imaged using an LSM 700 confocal microscope (Zeiss). Whole body embryo images were captured with a M205 FA wholemount epifluorescence microscope (Leica). Cell number quantification and pancreas length measurements were performed using ImageJ software^54^.

### Western blot analysis

Cell lysate from whole embryos was prepared by homogenization (Polytron PT2100) in lysis buffer (50 mM Tris (pH 8.0), 150 mM NaCl, 0.05% Desoxycholate, 0.1% IGEPAL, 0.1% SDS, 0.2% sarcosyl, 10% glycerol, 1 mM DTT, 1mM EDTA, 10 mM Na, protease inhibitors (complete Mini, EDTA-free, Roche), phosphatase inhibitors (PhosStop, Roche), 2 mM MgCl_2,_ 0.05% v/v Benzonase) followed by western blot analysis. The antibodies used for detection of protein include mouse anti-deoxyhypusine synthase (Santa Cruz), rabbit anti-eIF5A^Hyp^^30^, and rabbit anti-Erk1/2 (Cell Signaling). IRDye and VRDye conjugated secondary antibodies were used for visualization (LI-COR).

### Proliferation assay

5-ethynyl-2′-deoxyuridine (EdU) was injected into each embryo at 72 hpf by cardiac puncture using a microinjector (Toohey Company). EdU was permitted to incorporate into DNA for 30 minutes. The Click-iT EdU Alexa Fluor 647 Imaging Kit (Invitrogen) was used to detect EdU. Embryos were counterstained with DAPI and whole pancreas Z-stack images were obtained using a LSM 700 confocal microscope (Zeiss). The percentage of proliferating cells was determined by evaluating the number of EdU+/GFP + cells as a function of the total number of GFP + cells at the midline of the pancreas.

### Statistical analysis

Data are presented as the mean ± SEM. One-way ANOVA with Tukey’s Multiple Comparison Test was used for comparisons involving more than two conditions, and a two-tailed Student *t* test was used for comparisons involving two conditions. In all cases, significance was indicated on the graph as follows: *p* ≤ 0.05 (*), *p* ≤ 0.01 (**), *p* ≤ 0.001 (***). Prism5 software (GraphPad) was used for all statistical analyses.

## Additional Information

**How to cite this article**: Mastracci, T. L. *et al.* Polyamine biosynthesis is critical for growth and differentiation of the pancreas. *Sci. Rep.* **5**, 13269; doi: 10.1038/srep13269 (2015).

## Supplementary Material

Supplementary Information
