# Supplementary material for: Polyamine biosynthesis is critical for growth and differentiation of the pancreas
Source: Sci Rep. 2015 Aug 24;5:13269. doi: 10.1038/srep13269 (PMC4547391; doi:10.1038/srep13269)
Supplement: Supplementary Information [file srep13269-s1.pdf]

SUPPLEMENTARY FIGURES AND LEGENDS

‘Polyamine biosynthesis is critical for growth and differentiation of the pancreas’

Teresa L. Mastracci, Morgan A. Robertson, Raghavendra G. Mirmira and Ryan M. Anderson

Supplementary Fig. S1. Protein alignment of Odc1.

Comparison of human, mouse and zebrafish Odc1 protein sequence.

```
human_ODC1   MNNFGNEEFDCHFLDEGFTAKDILDQKINEVSSSDDKDAFYVADLGDILKKHLRWLKALP
mouse_Odc1   MSSFTKDEFDCHILDEGFTAKDILDQKINEVSSSDDKDAFYVADLGDILKKHLRWLKALP
zfish_Odc1   MTACTGSDFDFAFLEEGFCARDIVEQKINESSLSDDKDAFYVADLGDVLKKHLRWLRVLP
             *.    .:**  :*:*** *:**::***** * **************:********:.**

human_ODC1   RVTPFYAVKCNDSKAIVKTLAATGTGFDCASKTEIQLVQSLGVPPERIIYANPCKQVSQI
mouse_Odc1   RVTPFYAVKCNDSRAIVSTLAAIGTGFDCASKTEIQLVQGLGVPAERVIYANPCKQVSQI
zfish_Odc1   RITPFYAVKCNDSRAVVTTLASLGAGFDCASKTEIQIVQSVGVDPSRIIYANPCKQVSQI
             *:***********:*:*.***: *:***********:**.:** ..*:************

human_ODC1   KYAANNGVQMMTFDSEVELMKVARAHPKAKLVLRIATDDSKAVCRLSVKFGATLRTSRLL
mouse_Odc1   KYAASNGVQMMTFDSEIELMKVARAHPKAKLVLRIATDDSKAVCRLSVKFGATLKTSRLL
zfish_Odc1   KYASAHGVQMMTFDSEVELMKVARSHENAKLVLRIATDDSKAVCRLSVKFGATLKSSRLL
             ***: :**********:*******:* :**************************::****

human_ODC1   LERAKELNIDVVGVSFHVGSGCTDPETFVQAISDARCVFDMGAEVGFSMYLLDIGGGFPG
mouse_Odc1   LERAKELNIDVIGVSFHVGSGCTDPETFVQAVSDARCVFDMATEVGFSMHLLDIGGGFPG
zfish_Odc1   LERAKELGLDVIGVSFHVGSGCTDPETYSQAISDARYVFDIGAELGYNMSLLDIGGGFPG
             *******.:**:***************: **:**** ***:.:*:*:.* **********

human_ODC1   SEDVKLKFEEITGVINPALDKYFPSDSGVRIIAEPGRYYVASAFTLAVNIIAKKIVLKEQ
mouse_Odc1   SEDTKLKFEEITSVINPALDKYFPSDSGVRIIAEPGRYYVASAFTLAVNIIAKKTVWKEQ
zfish_Odc1   SEDTKLKFEEIAAVINPALDKYFPVDSGVRIIAEPGRYYVASAYTLAVNIIAKKVIMKEQ
             ***.*******:.*********** ******************:********** : ***

human_ODC1   TGSDDE-DESSEQTFMYYVNDGVYGSFNCILYDHAHVKPLLQKRPKPDEKYYSSSIWGPT
mouse_Odc1   PGSDDE-DESNEQTFMYYVNDGVYGSFNCILYDHAHVKALLQKRPKPDEKYYSSSIWGPT
zfish_Odc1   SASDEEEDVSNDRTLMYYVNDGVYGSFNCILYDHAHVLPTLHKKPKPDERMYPCSIWGPT
             ..**:* * *.::*:********************** . *:*:*****: *..******

human_ODC1   CDGLDRIVERCDLPEMHVGDWMLFENMGAYTVAAASTFNGFQRPTIYYVMSGPAWQLMQQ
mouse_Odc1   CDGLDRIVERCNLPEMHVGDWMLFENMGAYTVAAASTFNGFQRPNIYYVMSRPMWQLMKQ
zfish_Odc1   CDGLDRIVEQCSLPDMQVGDWLLFENMGAYTVAASSTFNGFQKPDIYYIMSRTAWQCMQQ
             *********:*.**:*:****:************:*******:* ***:** . ** *:*

human_ODC1   FQNPDFPP-EVEEQDASTLPVSCAWESGMKRHRAACASASINV
mouse_Odc1   IQSHGFPP-EVEEQDDGTLPMSCAQESGMDRHPAACASARINV
zfish_Odc1   IRAQGIPALPLEEPSAGNVPSHCGRESSLDVPAKPCPTQVL--
             ::  .:*.  :** . ..:*  *. **.:.    .*.:  :
```

Supplementary Fig. S2. Titration of DFMO in zebrafish.

(a) Percentage of viable embryos in control (n = 180), 1% w/v DFMO-treated (n = 33), 2% w/v DFMO-treated (n = 45), 3% w/v DFMO-treated (n = 28), 5% w/v DFMO-treated (n = 24), and 10% w/v DFMO-treated (n = 15) embryos at 72 hpf. (b) Percentage of control (n = 32), 1% w/v DFMO-treated (n = 33), 2% w/v DFMO-treated (n = 40) embryos at 72 hpf with altered body axis due to DFMO treatment. (c) Representative images of control and DFMO-treated *Tg(ptf1a:gfp)* embryos displaying the phenotype of altered (truncated) body axis. (d) The *ptf1a*: GFP-expressing retina (r) and hindbrain (hb) were unaltered in DFMO-treated *Tg(ptf1a:gfp)* embryos compared with controls, at 48 hpf and 72 hpf.

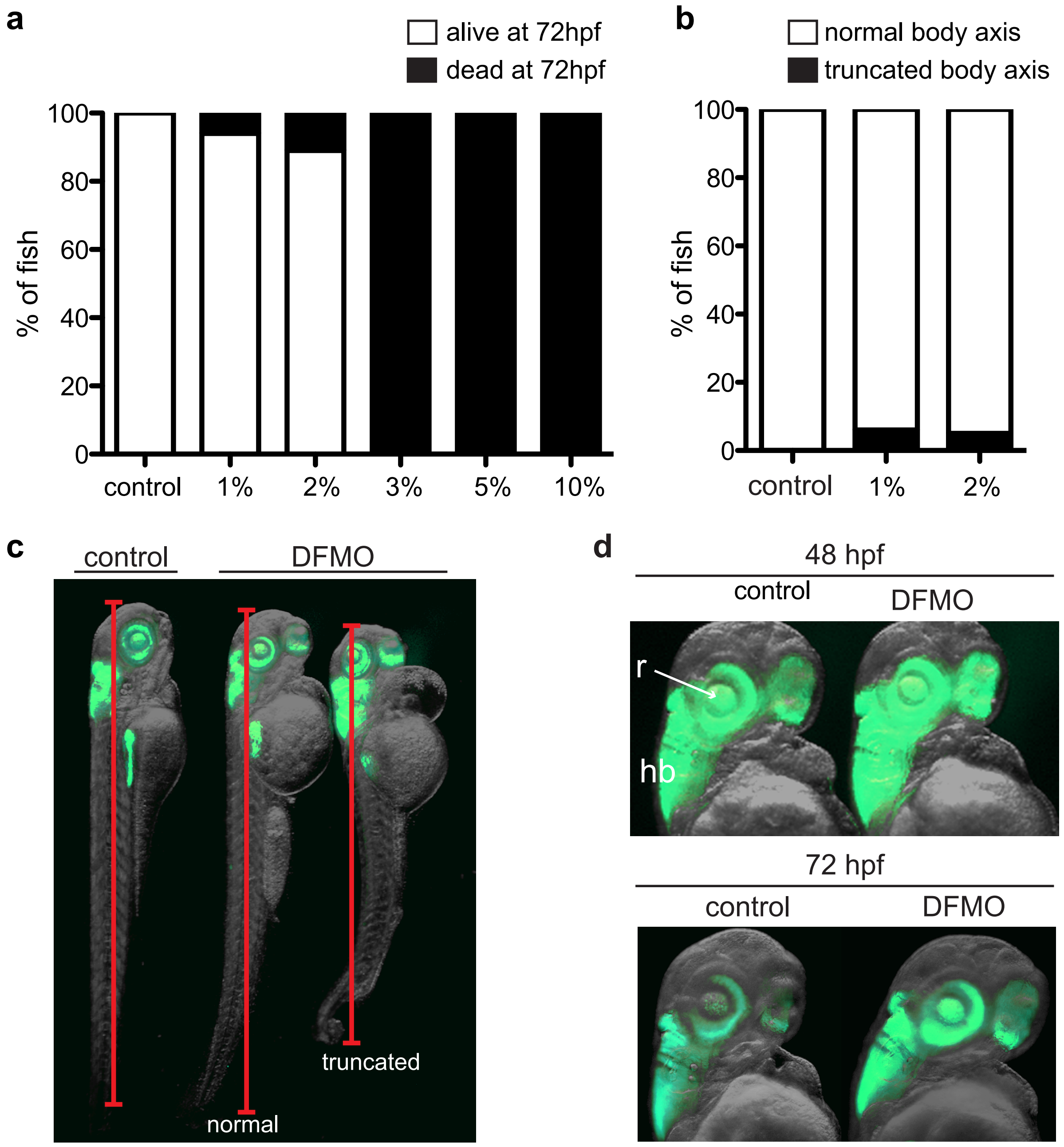

Supplementary Fig. S3. Endocrine cell phenotype following inhibition of polyamine biosynthesis by DFMO treatment.

Representative images at 72 hps of glucagon-expressing cells in (a) control and (b) DFMO-treated *Tg*(*ptf1a:gfp*) embryos; insulin-expressing cells in (c) control and (d) DFMO-treated *Tg*(*ptf1a:gfp*) embryos; and insulin/glucagon co-expressing cells in (e) control and (f) DFMO-treated *Tg*(*ptf1a:gfp*) embryos. (g) Glucose measurement from control or DFMO-treated embryos at 72 hpf (n = 3 groups of 10 embryos/group) (p = 0.4821). (h) Western blot analysis for expression of DHS, $eIF5A^{Hyp}$, and Erk1/2 in control and DFMO-treated embryos at 72 hpf (n = 4 groups of 30 embryos/group); Erk1/2 was used as a loading control, as expression of this protein is not altered with DFMO treatment. Quantification showed no change in (i) DHS expression (p = 0.2370) but a significant decrease in (j) $eIF5A^{Hyp}$ expression (p = 0.0039). gluc, glucagon; ins, insulin. * p < 0.05, ** p < 0.01, *** p < 0.001; ns, not significant.

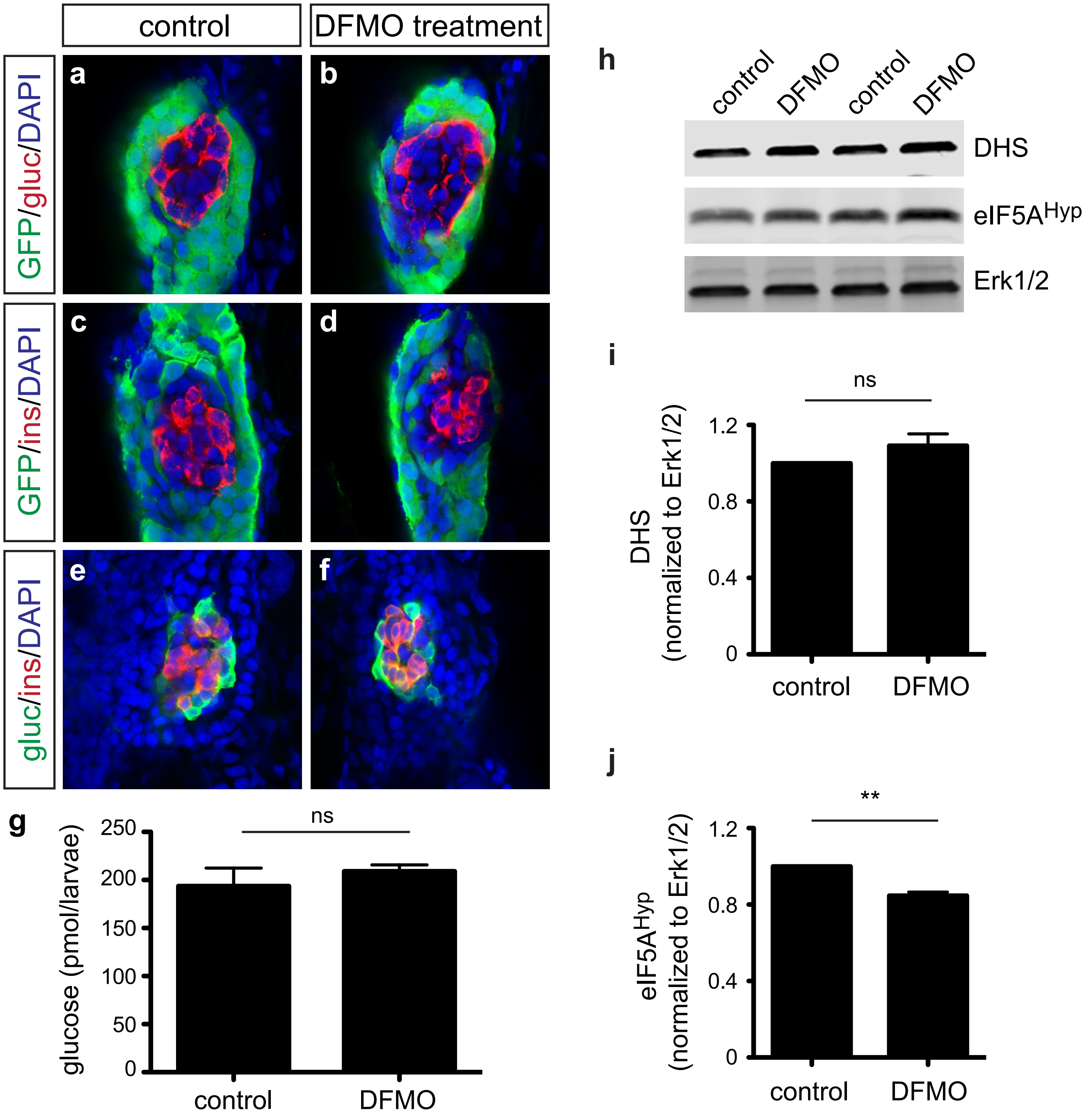

Supplementary Fig. S4. Protein alignment of DHS and Eif5a.

Comparison of human, mouse and zebrafish (a) Dhps and (b) Eif5a protein sequence. The K50 residue of Eif5a is highlighted in red, and is the residue hypusinated by DHS.

a

```
human_DHPS    MEGSLEREAPAGALAAVLKHSSTLPPESTQVRGYDFNRGVNYRALLEAFGTTGFQATNFG
mouse_Dhps    MEGTPPGAAPSSALAAVLKHSSALPPESAQVQGYDFNRGVDYHALLDAYRTTGFQATNFG
zfish_Dhps    ----MAGQAPPVARAAVLKESTPLPDNLPQIRGYDFNQGLNHRALLQSFITTGFQASSFG
                      **  * *****.*: ** :  *::*****:*::::***::: ******:.**

human_DHPS    RAVQQVNAMIEKKLEPLSQDEDQHADLTQSRRPLTSCTIFLGYTSNLISSGIRETIRYLV
mouse_Dhps    RAVQQVNAMIEKKLEPLAVDEDHHADLTQSRRPLTGCTIFLGYTSNLISSGIRETIRYLV
zfish_Dhps    LAVQEINKMIEKRLEPVQEECE----DSDSRQSALGCTIFLGYTSNLISSGVRESIRYLT
               ***::* ****:***:  : :     ::**:   .***************:**:****.

human_DHPS    QHNMVDVLVTTAGGVEEDLIKCLAPTYLGEFSLRGKELRENGINRIGNLLVPNENYCKFE
mouse_Dhps    QHNMVDVLVTTAGGVEEDLIKCLAPTYLGEFSLRGKELRESGINRIGNLLVPNDNYCKFE
zfish_Dhps    QHKMVDVIVTTAGGIEEDLIKCLAPTFLGEFSLKGKELRQQGINRIGNLLVPNENYCKFE
              **:****:******:***********:******:*****:.************:******

human_DHPS    DWLMPILDQMVMEQNTEGVKWTPSKMIARLGKEINNPESVYYWAQKNHIPVFSPALTDGS
mouse_Dhps    DWLMPILDQMVLEQNTEGVKWTPSKMISRLGKEINNPDSVYYWAHKNHIPVLSPALTDGS
zfish_Dhps    DWLMPILDQMVLEQKTEGTHWTPSKMIHRLGKEINNPDSVYYWAYKNDIPVFSPALTDGS
              ***********:**:***.:******* *********:****** **.***:********

human_DHPS    LGDMIFFHSYKNPGLVLDIVEDLRLINTQAIFAKCTGMIILGGGVVKHHIANANLMRNGA
mouse_Dhps    LGDMIFFHSYKNPGLVLDIVEDLRLINTQAIFAKRSGMIILGGGVVKHHIANANLMRNGA
zfish_Dhps    LGDMIYFHSYKNPGLVLDIVEDIRRLNSKAVFAKSTGMIILGGGLVKHHIANANLMRNGA
              *****:****************:* :*::*:*** :********:***************

human_DHPS    DYAVYINTAQEFDGSDSGARPDEAVSWGKIRVDAQPVKVYADASLVFPLLVAETFAQKMD
mouse_Dhps    DYAVYINTAQEFDGSDSGARPDEAVSWGKIRMDAQPVKVYADASLVFPLLVAETFAQKAD
zfish_Dhps    DFAVFVNTGQEFDGSDSGARPDEAISWGKIRMDASPVKVYADASLVFPLLVAETFAHNAS
              *:**::**.***************:******:**.*********************:: .

human_DHPS    AFMHEKNED
mouse_Dhps    AFRAEKNED
zfish_Dhps    RLIQEKKSD
               :  **:.*
```

b

```
human_EIF5A   -MADDLDFETGDAGASATFPMQCSALRKNGFVVLKGRPCKIVEMSTSKTGKHGHAKVHLV
mouse_Eif5a   -MADDLDFETGDAGASATFPMQCSALRKNGFVVLKGRPCKIVEMSTSKTGKHGHAKVHLV
zfish_Eif5a   MADPDLDFASGDAGASATYPMQCSALRKNGFVVLKGRPCKIVEMSTSKTGKHGHAKVHLV
                  **** :********:*****************************************

human_EIF5A   GIDIFTGKKYEDICPSTHNMDVPNIKRNDFQLIGIQDGYLSLLQDSGEVREDLRLPEGDL
mouse_Eif5a   GIDIFTGKKYEDICPSTHNMDVPNIKRNDFQLIGIQDGYLSLLQDSGEVREDLRLPEGDL
zfish_Eif5a   GIDIFTNKKYEDICPSTHNMDVPNIKRNDYQLVDISEGFLSLMMDNGDVREDLRVPDGDL
              ****** **********************:**: *.:*:***: *.*:******:*:***

human_EIF5A   GKEIEQKYDCGEEILITVLSAMTEEAAVAIKAMAK
mouse_Eif5a   GKEIEQKYDCGEEILITVLSAMTEEAAVAIKAMAK
zfish_Eif5a   GKEIENKFAAGEEMLVTVLSAMGEESAVALKPMTK
              *****:*: .***:*:****** **:***:* *:*
```

Supplementary Fig. S5. Expression patterns of *eif5a* in zebrafish at 48 hpf.

At 48 hpf, (a) *eif5a* has strong expression in the exocrine compartment compared with the (b) *neurod1*:GFP+ endocrine cell compartment; (c) shows the merged image. (d) Image showing the pancreas (dotted box) and adjacent neural tube. (e) The corresponding maximum intensity projection of *eif5a* expression in the pancreas at 48 hpf; dotted box denotes pancreas domain.

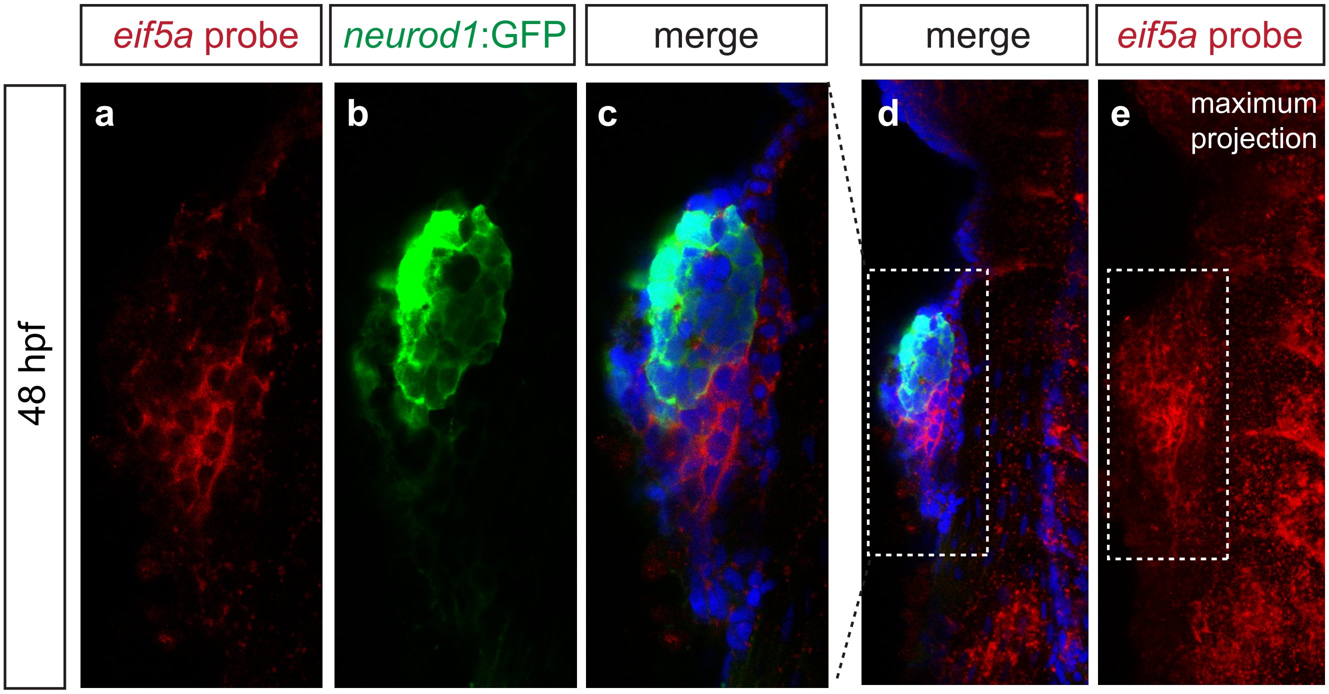

Supplementary Fig. S6. Phenotypic penetrance and expressivity in *dhps* MO-injected embryos.

(a) Control, 1 ng MO-injected, and 2 ng MO-injected *Tg(ptf1a:gfp)* embryos displaying the phenotype of normal or altered (truncated) body axis. White dotted box demarks pancreas.
(b) Percentage of control (n = 20), 1 ng MO-injected (n = 46), and 2 ng MO-injected (n = 23) embryos with altered body axis following implementation of the *dhps* MO.
(c) Sequencing data showing the deletion of exon 2 as a result of *dhps* MO knockdown. Primers used for PCR amplification are highlighted in green; the deleted region is indicated by dashed lines.

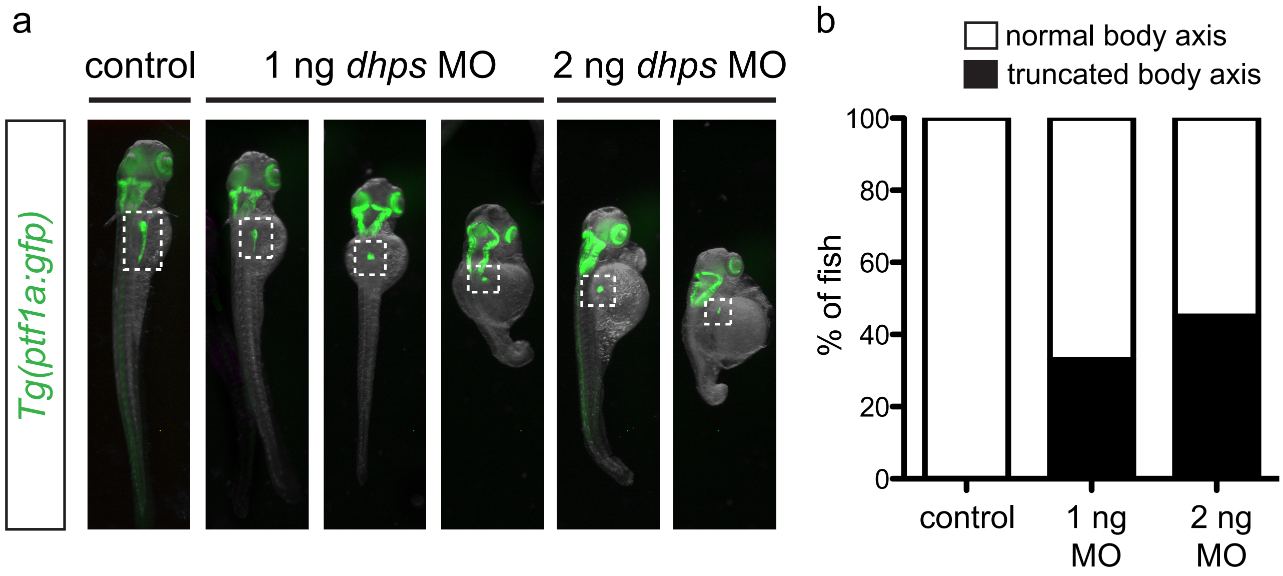

c

```
zf dhps cDNA GCGCTGTGAAATGTGAGTGAAACCAACGAGAAACTACACCTAAAAAGCCGG
zf dhps MO   GCGCTGTGAAATGTGAGTGAAACCAACGAGAAACTACACCTAAAAAGCCGG
             ***************************************************

zf dhps cDNA ATTTACATCCATAACCCCCGTGCGGATCGGTGAGCTCCTCATGGCGGGTCA
zf dhps MO   ATTTACATCCATAACCCCCGTG-----------------------------
             **********************-----------------------------

zf dhps cDNA GGCTCCGCCGGTGGCCCGGGCCGCGGTGCTGAAGGAGAGCACGCCTCTGCC
zf dhps MO   ---------------------------------------------------
             ---------------------------------------------------

zf dhps cDNA GGACAATCTGCCTCAGATCAGAGGATACGACTTTAACCAGGGCCTGAACCA
zf dhps MO   ---------------------------------------------------
             ---------------------------------------------------

zf dhps cDNA CAGAGCGCTGCTGCAGTCCTTCATCACTACCGGCTTCCAGGCCTCCAGCTT
zf dhps MO   ---------------------------------------------------
             ---------------------------------------------------

zf dhps cDNA CGGCCTGGCAGTGCAGGAGATCAATAAGATGATAGAGAAGAGGCTGGAGCC
zf dhps MO   -------------------------------ATAGAGAAGAGGCTGGAGCC
             -------------------------------********************

zf dhps cDNA GGTGCAGGAGGAGTGTGAGGACAGTGATTCTCGTCAGTCTGCTTTGGGATG
zf dhps MO   GGTGCAGGAGGAGTGTGAGGACAGTGATTCTCGTCAGTCTGCTTCGGGATG
             ***************************************************

zf dhps cDNA CACTATATTCCTGGGCTACACGTCAAAC
zf dhps MO   CACTATATTCCTGGGCTACACGTCAAAC
             ****************************
```
